# Ambient-densified and polymer-free transparent wood film for smart food packaging window

**DOI:** 10.1016/j.isci.2023.108455

**Published:** 2023-11-15

**Authors:** Kailong Zhang, Isaac Sutton, Micholas Dean Smith, David P. Harper, Siqun Wang, Tao Wu, Mi Li

**Affiliations:** 1 Center for Renewable Carbon, School of Natural Resources, University of Tennessee, Knoxville, TN 37996, USA; 2 Department of Chemical and Biomolecular Engineering, University of Tennessee, Knoxville, TN 37996, USA; 3 Department of Biochemistry and Cellular and Molecular Biology, University of Tennessee, Knoxville, TN 37996, USA; 4 Department of Food Science, University of Tennessee, Knoxville, TN 37996, USA

**Keywords:** Biotechnology, biomass, materials science, materials chemistry, Biomaterials

## Abstract

Wood, with its inherent hierarchical structure, presents opportunities for creating eco-friendly and cost-effective alternatives to petroleum-based plastics. We introduced a top-down and polymer-free method for engineering natural balsa wood into transparent wood film, demonstrating its potential use in food packaging windows. The wood was delignified and then proceeded with 2,2,6,6-tetramethyl-1-piperidinyloxy oxidation to soften the wood structure and introduce carboxyl groups. A robust and transparent wood film was produced by drying the wood under ambient condition without the need for additional polymers or mechanical force. Curcumin was also integrated into the wood using vacuum impregnation. The functionalized wood film with curcumin (WFC) exhibited a distinguishable redness shift in alkaline conditions. We then applied the WFC as an intelligent food packaging window to sense the freshness of shrimp based on the pH-responsive color change. This study provides a simple and scalable approach for developing sustainable and smart food packaging using wood.

## Highlights

• Transparent wood film was fabricated by delignification, oxidation, and air-drying
• No external polymers or mechanical forces were used in the film fabrication process
• The curcumin-functionalized film enabled a smart window to monitor freshness for food packaging

## Graphical abstract

**Figure:**
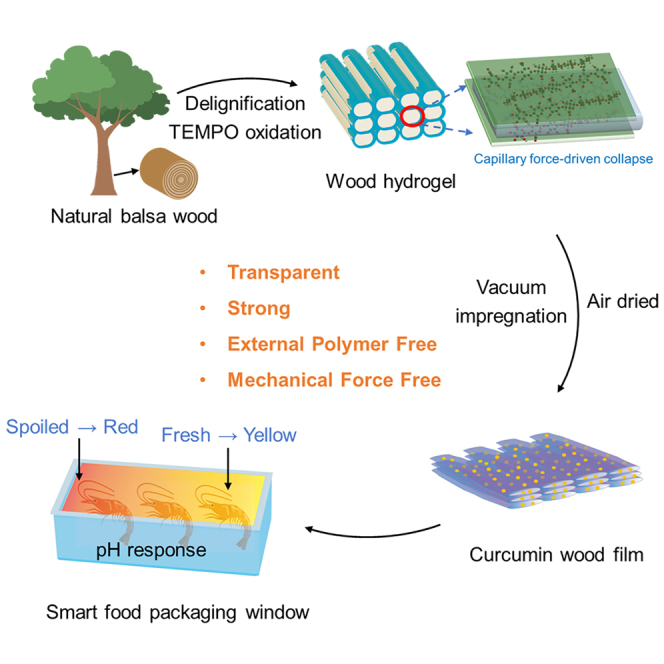

## Introduction

Wood, a widely available renewable resource, covers over 30% of the Earth’s land area and has been used extensively in construction, fuel, furniture, and tools. With the goals of reducing carbon footprint and leveraging advancements in nanotechnology, wood-based functional materials have been developed for multiple applications, such as water purification, energy harvesting, electronics, optics, nanofluidics, and fire-retardant materials.^1^^,^^2^^,^^3^ In particular, the discovery of transparent wood within the past two decades has significantly broadened the scope of wood-based functional materials and their potential applications.^4^

Transparent wood can be fabricated using bottom-up or top-down strategies. The bottom-up method involves disintegrating the wood cell wall matrix into nanoscale cellulosic materials, like cellulose nanocrystals (CNCs) or cellulose nanofibers (CNFs), which are then employed as precursors to construct transparent cellulosic materials. However, this approach requires significant mechanical energy input or chemical reagents and is complex and labor-intensive.^5^^,^^6^ The low energy efficiency and high cost of this bottom-up method pose substantial challenges for scaling up the production of transparent wood-based materials. Furthermore, this method deconstructs the innate three-dimensional hierarchical structure characteristic and the fiber orientation of wood upfront. Alternatively, the top-down strategy circumvents the process for nanocellulose isolation and reconstruction by manipulating and modifying natural wood structure to achieve transparency and other functions. This approach typically begins with delignification of wood to remove chromophoric lignin and extractives, resulting in porous and cellulose-rich wood. Subsequently, the process involves infiltrating the wood with polymers that have a refractive index matching that of cellulose to create transparent wood materials or, alternatively, using densification to remove air traps that reduce light scattering to produce transparent films.^6^^,^^7^^,^^8^ This top-down approach offers superior advantages over the bottom-up approach in two key aspects: (i) it requires less time, fewer chemicals, and reduced energy consumption and (ii) it effectively maintains the cellulose orientation and hierarchical structure of wood.^9^^,^^10^^,^^11^^,^^12^^,^^13^ As a result, the top-down approach represents a promising and straightforward technique for the mass production of transparent wood-based materials.

Recently, transparent wood has been creatively explored for sensing and sensor applications. Highly stretchable and electrically conductive transparent wood has been developed by backfilling delignified wood with polymerizable deep eutectic solvents, using a combination of acrylic acid and choline chloride. This process imparted excellent stretchability to the transparent wood, resulting in exceptional sensing behavior for strain and pressure.^14^ Another study showed that the electrical conductivity of transparent wood responded to temperature changes.^15^ By encapsulating carbon dots with varying fluorescent detection sensitivity within a delignified wood framework, luminescent transparent wood can be created to respond and monitor indoor formaldehyde pollutants.^16^ Furthermore, transparent wood film (WF) served as a flexible and robust substrate for printing conductive ink, forming a flexible electronic circuit for strain sensing.^17^ By sandwiching two transparent WFs as conductive electrodes and a stimuli-responsive polydimethlysiloxane layer, a wood-based electronic skin with high capacitive sensitivity was fabricated.^18^ Transparent and functional wood has also been explored for its potential in packaging applications. A fully biobased transparent wood with great properties for packaging has been developed by infiltrating CNFs and chitosan into the pores of the bleached Fir veneer wood using a vacuum jar.^19^ Similarly, incorporating poly(methyl methacrylate) and UV/visible light switchable photochromic dyes into delignified wood leads to photochromic transparent wood-based packaging with UV-shielding capabilities and switchable transmittance and color.^20^ While these studies present remarkable advancements in the field, they commonly rely on the incorporation of external polymers into the wood structure for enhanced functionality.

As an alternative to the polymer impregnation method, densification of delignified wood represents a crucial and straightforward approach to creating transparent films. By applying external pressure to delignified wood, light scattering within the porous wood is significantly reduced, resulting in enhanced light transmittance, while the highly aligned cellulose fibers are preserved. This simple method leads to wood-based transparent films with anisotropic microstructures and exceptional mechanical strength,^21^ flexible electronics,^17^ luminescent and hydrophobic properties,^22^ as well as high-performance capabilities and biodegradability.^23^ The crucial role of water molecules in strengthening hydrogen bonding between cellulose fibers, which leads to robust and resilient structural materials, was demonstrated by regulating the moisture content during the compression and drying processes.^24^ Capitalizing on the power of hydrogen bonding, a pioneering top-down technique has been introduced for producing transparent WFs without the need for external pressure or additional polymer infiltration.^25^ The delignified wood was first oxidized using 2,2,6,6-tetramethyl-1-piperidinyloxy (TEMPO) to introduce carboxylic groups to the mesoporous cell walls. Then the transparent film was obtained with remarkable mechanical strength by simply self-densifying the oxidized wood in air. Cellulose’s numerous carboxyl and hydroxyl groups tend to form robust hydrogen bonds, resulting in the densification of the wood cell walls.^25^ This judicious discovery facilitates the creation of cost-effective, transparent, and uniquely functional wood-based films. Motivated by these advancements, we are designing self-densifying transparent WF sensors as a sustainable alternative for intelligent food packaging applications.

In this study, we have engineered an innovative wood-based transparent food packaging film with sensing capabilities using a simple top-down self-densification method under ambient conditions. Natural balsa wood was first delignified followed by TEMPO oxidation. Curcumin, a pH-responsive color indicator used in food packaging, was integrated into the WF through vacuum impregnation. The functionalized wood was then air-dried, allowing it to self-densify into films without the need for external polymers infiltration or additional mechanical forces. Inverse gas chromatography (iGC) was conducted to examine the surface energy profiles and elucidate the interactions occurring at the wood fiber interface. The functionalized wood film with curcumin (WFC) exhibited a pH-responsive color change capacity that can be used as a “smart window” to monitor the condition of food freshness.

## Results and discussion

### Characterization of delignified, oxidized, and self-densified wood

**Figure 1:**
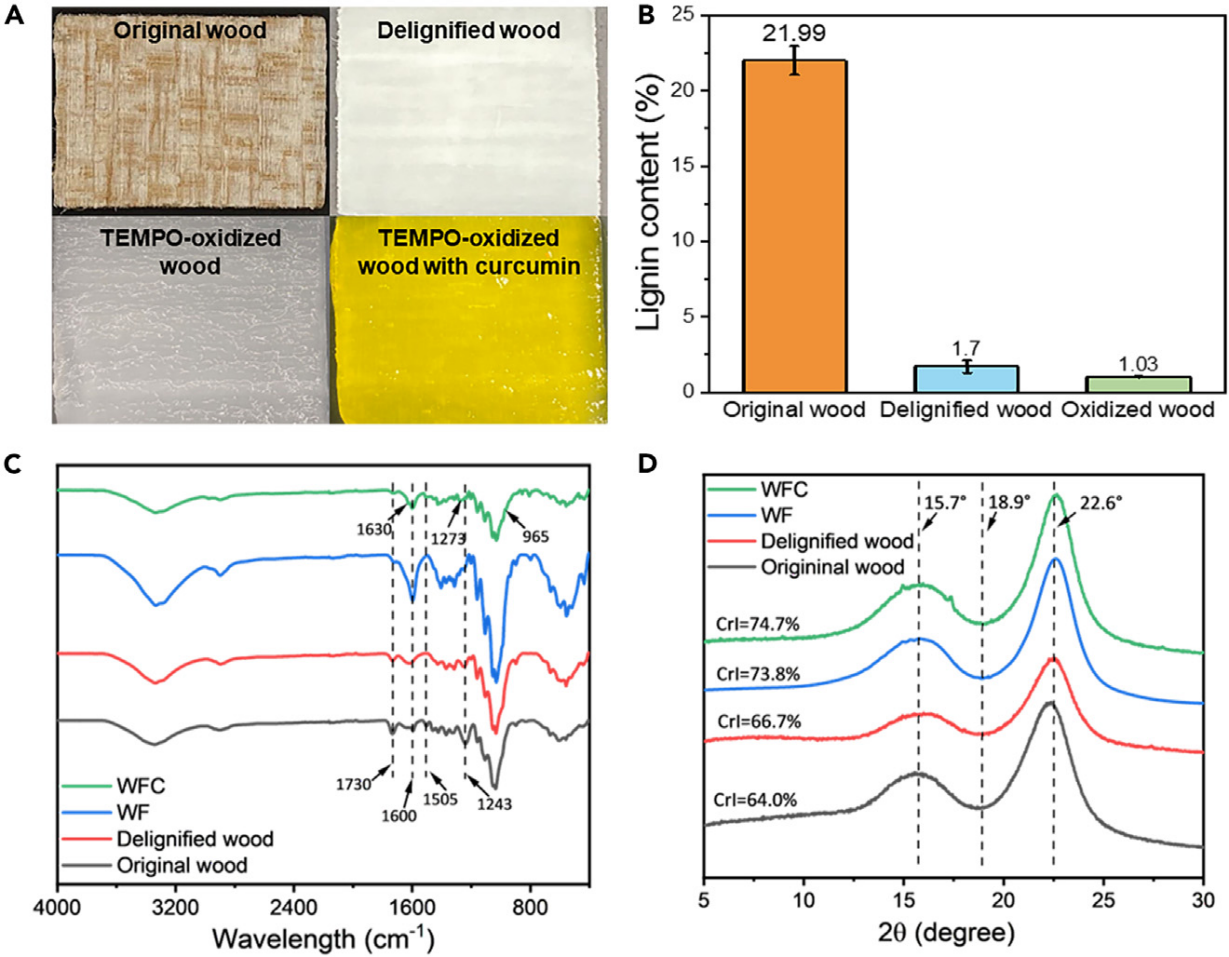
Visual appearance, lignin content, and chemical structure of original wood, delignified wood, TEMPO-oxidized wood, and curcumin-infused TEMPO-oxidized wood (A) Visual appearance of the original wood, delignified wood, TEMPO-oxidized wood hydrogel, and curcumin-infused TEMPO-oxidized wood hydrogel. (B) Acid-insoluble lignin content in the original wood, delignified wood, and TEMPO-oxidized wood. Data are represented as mean ± SEM. (C) ATR-FTIR spectra of the original wood, delignified wood, wood film (WF), and wood film with curcumin (WFC). (D) XRD patterns of the original wood, delignified wood, wood film (WF), and wood film with curcumin (WFC).

**Figure 2:**
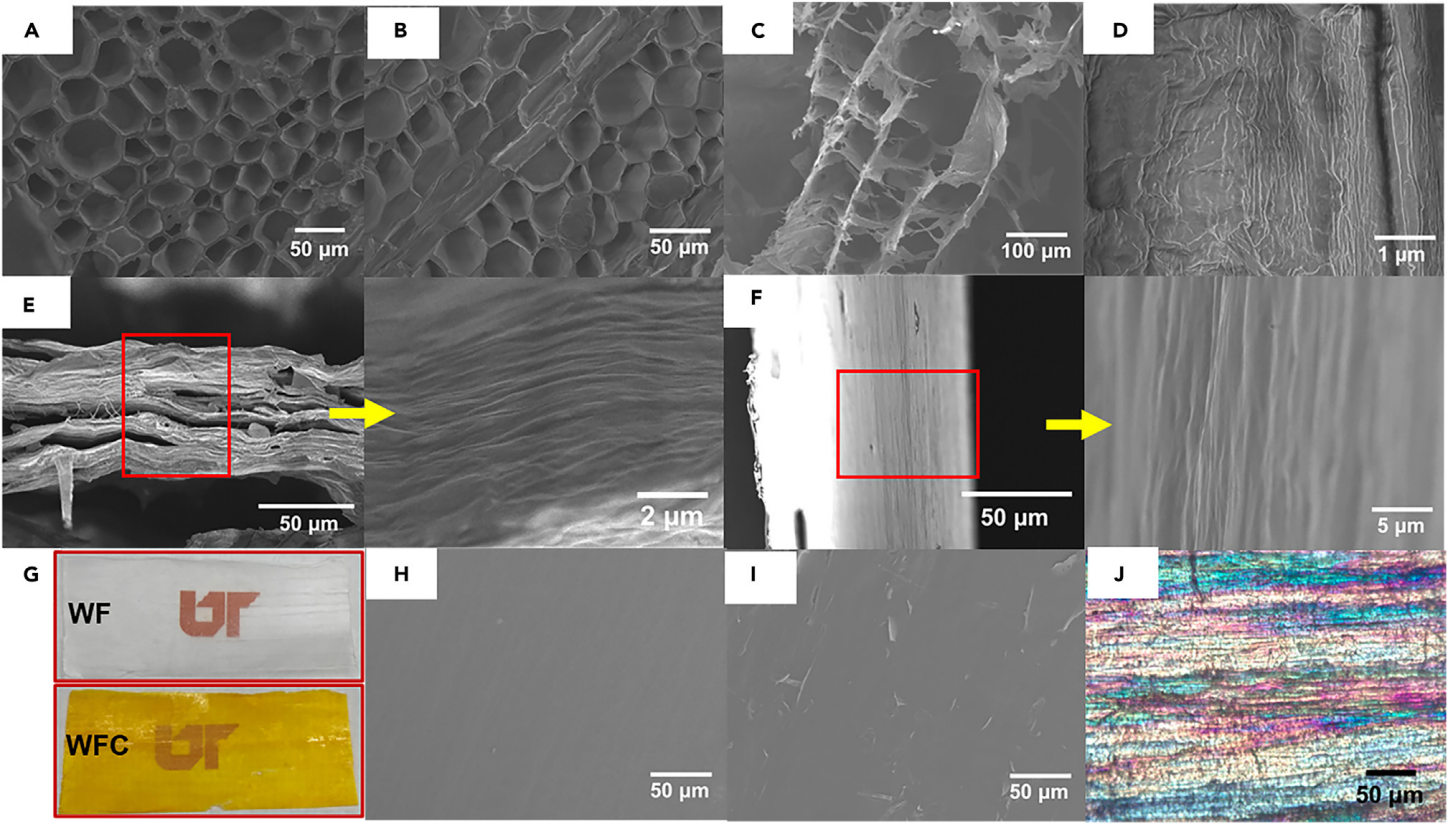
Morphology examination of original wood, delignified wood, TEMPO-oxidized wood, and wood films (A) SEM image of the cross-section of the original wood. (B) SEM image of the cross-section of the delignified wood. (C) SEM image of the cross-section of the TEMPO-oxidized wood. (D) SEM image of the longitudinal section of the TEMPO-oxidized wood. (E) SEM image of the cross-section of the densified wood film (WF) at two different magnifications. (F) SEM image of the longitudinal section of the densified wood film (WF) at two different magnifications. (G) Visual appearance of the wood film (WF) and wood film with curcumin (WFC). (H) SEM images of the surface of the wood film (WF). (I) SEM images of the surface of the wood film with curcumin (WFC). (J) Polarized light image of wood film (WF).

Wood is composed of paracrystalline cellulose fibril bundles, amorphous heteropolysaccharide hemicellulose, and polyphenol propane-based branched lignin. Lignin acts as a binder in wood tissue, filling in the gap of the cell wall and accounting for 15%–35% of the total dry mass.^26^ Partial or complete elimination of this cementing substance exposes the cellulose scaffolds, providing the feasibility to create functional materials based on cellulose.^9^ NaClO_2_ is commonly used in the bleaching process for delignification. In an acidic solution, chlorine dioxide gas generated from chlorous acid is the major active agent that oxidizes the phenolic structure of lignin to a phenoxyl radical. Further oxidation results in the formation of a monomethyl ester of the muconic acid derivative.^27^ Lignin is responsible for the brownish color of wood, which was attributed to chromophores such as double bond-aromatic ring conjugations and carbonyl bonds. These chromophores contribute to 80%–95% of light absorption in wood.^28^ The delignification process using NaClO_2_ effectively reduced the lignin content in balsa wood, resulting in white wood with significantly lower lignin content of 1.7% compared with the original 21.99% (Figures 1A and 1B). The effectiveness of the delignification process was demonstrated by the decreased Fourier transform infrared (FTIR) peak intensity at 1,730 and 1,243 cm^−1^, corresponding to the C=O and C-O groups, respectively, and the disappearance of the peak at 1,505 cm^−1^ corresponding to the lignin aromatic ring skeleton vibration (Figure 1C).^29^ A previous study observed a dramatic decline in lignin (from 24.9% to 2.9%) in balsa wood after bleaching with NaClO_2_.^30^ Despite the substantial delignification, the hierarchical alignment and micro-porous structure (lumen size of 30–60 μm) of the wood were well preserved with minimal damage compared with the original wood (Figures 2A and 2B).

TEMPO-mediated oxidation has been widely used for the fabrication of carboxylated CNF. During oxidation, the -OH groups at the carbon six position of anhydroglucose units undergo selective conversion into carboxyl groups. A TEMPO/NaBr/NaClO combination at pH 10 is commonly employed in cellulose oxidation due to the mild reaction conditions conducted at room temperature. After oxidation, nanoscale cellulose fibrils with diameter of 3–4 nm can be exfoliated from the carboxylated cellulose bundles through mechanical disintegration.^31^ However, when applied to wood, the alkaline reaction conditions at pH 10 can partially dissolve the hemicellulose, compromising the structural integrity of the original wood. An alternative oxidation approach is to use the TEMPO/NaClO/NaClO_2_ reaction system at mildly acidic conditions (i.e., pH 4.8–6.8).^5^ In this work, delignified wood samples were oxidized using TEMPO/NaClO/NaClO_2_ under neutral conditions (pH 6.8) to preserve wood cell wall’s structural integrity. The TEMPO-oxidized wood hydrogel showed a translucent and gelatinous appearance (Figure 1A). The peak at 1,600 cm^−1^ in the FTIR spectra (Figure 1C), attributed to the carboxylate group, confirmed the oxidation of cellulose in the wood after TEMPO oxidation treatment. The concentration of carboxylic groups was determined to be 764 ± 11 μmol/g using the conductivity titration method. The micromorphology of freeze-dried wood aerogel showed that the wood cell structure was disrupted to some extent after the oxidation treatment, while the lumen cavities were largely maintained (Figure 2C). Compared with the alkaline TEMPO reaction condition with the TEMPO/NaBr/NaClO system at pH 10, we contend that the neutral reaction condition at pH 6.8 played an important role in preserving the structural integrity by reducing hemicellulose solubilization.^25^ The longitudinal view of the TEMPO-oxidized wood showed that cellulose fibrils in the cell wall formed well-aligned microfibrils with a width of tens of nanometers (Figure 2D) due to the electrostatic repulsion between the surface-carboxylated cellulose microfibrils.^32^ The lignin content of the TEMPO-oxidized wood was further reduced to 1.03% compared with the delignified wood of 1.7% (Figure 1B), attributed to the oxidation of remaining lignin macromolecules by NaClO_2_ in this process.

After the delignification and TEMPO oxidation, the strength and rigidity of the wood structure were undermined, resulting in soft and porous cellulose scaffolds (Figure 2C). The cellulose cell walls readily aggregated and stacked to form a compact WF driven by capillary force. During the dehydration of the WF, the abundant carboxylic acid groups generated from the TEMPO oxidation likely facilitated interfibrillar aggregation through hydrogen bonding. In addition, the capillary pressure within wood pores caused by the high surface tension of water induced imbalanced stress forces on the porous wall, leading to the collapse of the cell wall into laminates during water evaporation.^33^ The WF in this study had a thickness of approximately 50 μm, corresponding to a densification ratio of 30. A prior study reported a comparable densification ratio of 25 with a film by implementing the self-densification method on wood.^25^ The stack of multilayer cell walls could be discerned from both cross and longitudinal sections of the WF (Figures 2E and 2F), indicating the significant roles of fiber hornification and hydrogen bonding. The WF showed a smooth and dense surface from the top view after self-densification at room temperature (Figures 2G and 2H). The X-ray diffraction (XRD) analysis revealed that the crystalline patterns of the WF were similar to those of the pristine balsa wood and the delignified wood (Figure 1D). The peaks at 15.7° and 22.6° corresponded to the characteristic planes (110) and (200) of cellulose, respectively, indicating that no significant changes occurred in the cellulose crystals. However, the crystallinity index (CrI) of the WF sample had increased significantly, reaching 72.7%, as compared with the original balsa wood and delignified wood samples, which had CrI values of 64.0% and 66.7%, respectively. This notable increase in CrI was attributed to the delignification and TEMPO oxidation processes, which removed the amorphous lignin (majorly) and hemicellulose (minorly) for the WF sample.^19^ Additionally, polarized light microscopy revealed that the cellulose fibers were well aligned (birefringent characteristic, Figure 2J), suggesting that the natural orientation of cellulose in wood was retained during the entire process.

IGC is an effective tool for measuring the Brunauer-Emmett-Teller (BET) specific surface area in polymer and natural fibers with low surface areas.^34^ In this work, the specific surface area (*S*_*BET*_) for the original balsa wood was 2.67 m^2^/g (Table 1). The surface area of wood significantly increased to 5.25 m^2^/g after delignification. Furthermore, the TEMPO oxidation treatment increased the surface area of the wood to 7.02 m^2^/g. In this study, both delignified wood and TEMPO-oxidized wood were subjected to a freeze-drying process using water (i.e., without solvent exchange). Alternative dehydration methods, such as freeze-drying with *t*-BuOH or supercritical drying could lead to a significantly higher surface area for delignified wood, usually ranging from 10 to 40 m^2^/g.^35^ These alternative drying methods play a crucial role in preserving the wood’s structural integrity and preventing potential collapse due to the drying solvent’s low surface tension, which might explain the reported higher values of surface area compared with those in our study.

**Table 1:** BET specific surface area ($S_{BET}$), dispersive ($\gamma_{S}^{D}$), specific ($\gamma_{S}^{AB}$), and total surface energy ($\gamma_{S}^{Tot}$), and polarity index ($\gamma_{S}^{AB}$/ $\gamma_{S}^{Tot}$) at 30°C (mean value corresponding to 50% coverage)

	$S_{BET}$ (m^2^/g)	$\gamma_{S}^{D}$ mJ/m^2^	$\gamma_{S}^{AB}$ mJ/m^2^	$\gamma_{S}^{Tot}$ mJ/m^2^	$\gamma_{S}^{AB}$/ $\gamma_{S}^{Tot}$
Original wood	2.67	58.99	9.06	68.04	0.13
Delignified wood	5.25	48.11	4.56	52.24	0.09
TEMPO wood	7.02	44.28	2.84	47.05	0.06

Understanding the surface energy of the wood samples at different treatment stages can provide insights into the nature of surface functional groups and illuminate the interactions occurring between fibers within the cell wall during the densification process. All samples exhibited a significantly higher dispersive surface energy $\gamma_{S}^{D}$ relative to specific surface energy $\gamma_{S}^{AB}$ (Table 1), suggesting that dispersive surface energy (caused by dispersive forces) had a predominant influence on these wood fibers. It should be noted that the surface energy measured by iGC is defined as the excess energy at the surface of a material compared with the bulk. The surface energy of a material is determined by multiple factors including but not limited to the material’s chemical composition, surface structure, and environmental conditions. The delignification and TEMPO oxidation treatments significantly altered the chemical and physical properties of the wood through various aspects. Therefore, a direct comparison of surface energies between these samples is not appropriate due to the essential alteration of the material bulk and the inherent complexities of their structures and properties.^36^

**Figure 3:**
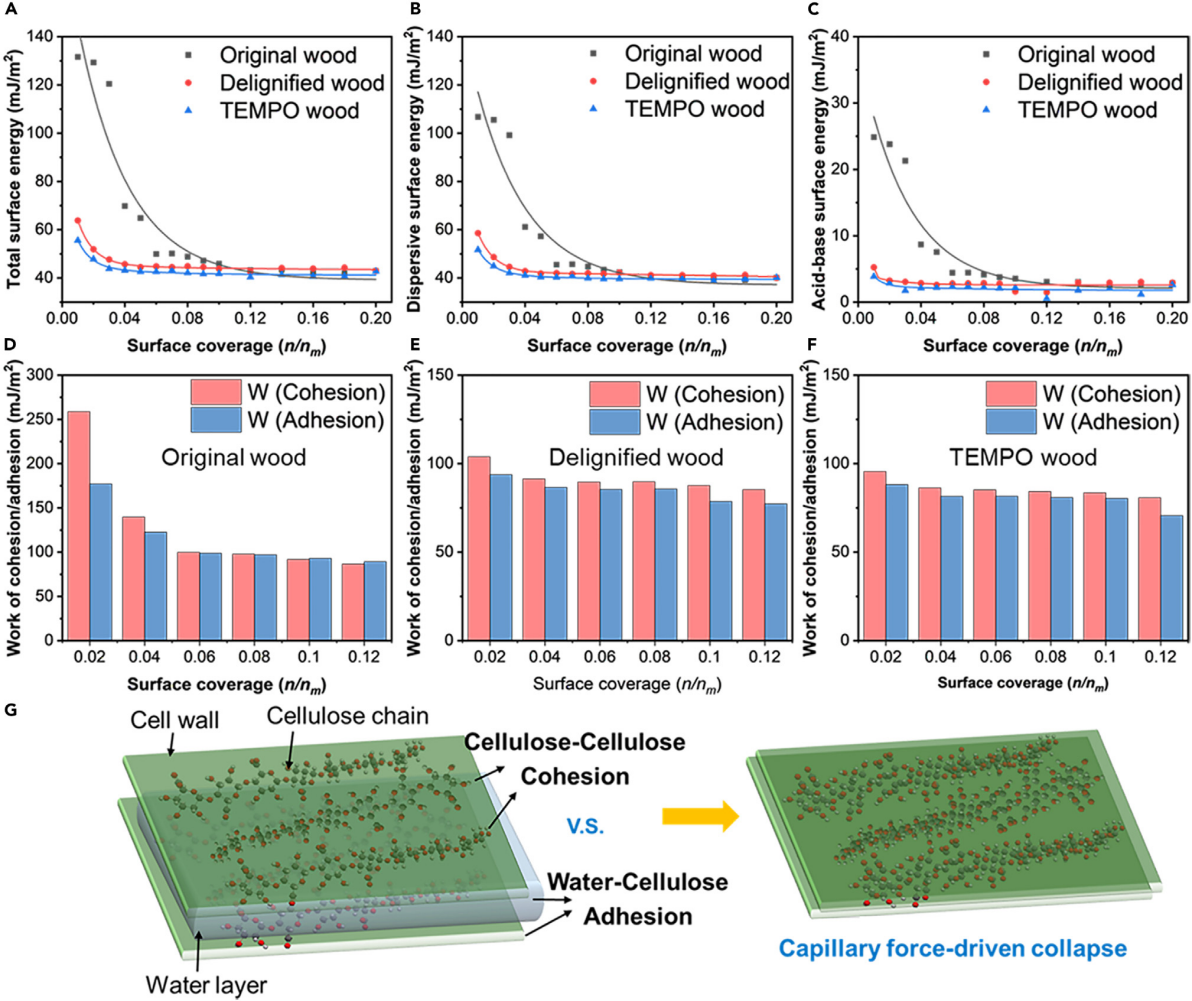
Surface energy evaluation of original wood, delignified wood, and TEMPO-oxidized wood See also Figure S1. (A) Total $\gamma_{S}^{Tot}$ surface energy. (B) Dispersive $\gamma_{S}^{D}$ surface energy. (C) Specific $\gamma_{S}^{AB}$ surface energy. (D) Work of cohesion within original wood and work of adhesion between original wood and water. (E) Work of cohesion within delignified wood and work of adhesion between delignified wood and water. (F) Work of cohesion within TEMPO-oxidized wood and work of adhesion between TEMPO-oxidized wood and water. (G) Representation of the capillary force-driven densification induced by the balance between cohesion and adhesion. Surface coverage is the number of moles (n) divided by the moles required to cover a surface monolayer (n_m_).

However, the variation of the surface energy after delignification and oxidation reflects the underlying chemical heterogeneity. The surface energy of the original wood exhibited notable heterogeneity as the surface coverage increased, while the delignified and TEMPO-oxidized wood exhibited a significantly reduced degree of surface energy heterogeneity across the entire tested coverage range (Figures 3A–3C). The untreated wood demonstrated the broadest distribution of dispersive and specific surface energy in comparison to both the delignified and TEMPO-oxidized wood, as illustrated in Figure S1. For delignified wood and TEMPO-treated wood, the enriched cellulosic component led to a correspondingly homogeneous and low-energy surface. This was corroborated by the lower total surface energy and narrow energy distribution observed. The dispersive surface energy is directly linked to the chemical heterogeneity present on the surface, rather than being impacted by surface roughness.^37^ Delignification and TEMPO oxidation processes reduced the lignin and extractive contents, thus enhancing the purity of cellulose. These treatments resulted in a reduction in the quantity of active sites on the surface and diminished chemical heterogeneity of the wood, leading to reduced dispersive surface energy. In addition, the decrease in lignin and extractives lowered the specific surface energy, partially because these compounds are rich in Lewis acid-base functional groups.^34^ The polarity index ($\gamma_{S}^{AB}$/ $\gamma_{S}^{Tot}$) decreased from 0.13 for the original wood to 0.09 for the delignified wood, and further to 0.06 for the TEMPO-oxidized wood, indicating the reducing proportion of the polar components following the processes.^38^

The thermodynamic work of adhesion and cohesion dictates the interaction strength at phase boundaries. The total work of cohesion ($W_{Coh}^{Tot}$) represents the inter-fiber attractive force, while the work of adhesion ($W_{Adh}^{Tot}$) corresponds to the interaction between fibers and water (Equations S1 and S2). The ratio of $W_{Adh}^{Tot}$/ $W_{Coh}^{Tot}$ indicates the balance of forces between water-fiber adhesion and fiber-fiber cohesion. An examination of this ratio provides insight into the comparative strength of these interactions.^39^ The original wood exhibited a relatively lower $W_{Adh}^{Tot}$/ $W_{Coh}^{Tot}$ ratio, especially at lower coverage, indicating a lower water affinity of the fiber. After the delignification and TEMPO oxidation processes, the overall $W_{Adh}^{Tot}$/ $W_{Coh}^{Tot}$ ratio typically increased (Figures 3D–3F), suggesting that the water-fiber adhesion force became stronger compared with the fiber-fiber cohesion force. As a result, capillary forces exerted greater influence within the system. The evaporation-induced capillary force then contributed significantly to the collapse of the wood cell wall and the facilitation of cellulose fiber densification (Figure 3G).^40^

### Curcumin-functionalized wood-based transparent films

The WFC was fabricated by vacuum impregnating the curcumin compounds into the wood’s pores and depositing them on the cell walls. Static penetration of additives into the wood cell is driven only by capillary forces, and diffusion is usually slow and typically restricted to a certain depth.^41^ The applied vacuum pressure facilitated the migration of curcumin molecules into the interior wood pores, as revealed by a thoroughly yellow wood hydrogel obtained after vacuum impregnation (Figure 1A). After the ambient self-densification process, a yellow WFC film with a thickness of 50 μm (same as that of WF without curcumin) was formed (Figure 2G). The surface of the WFC remained smooth after impregnation with curcumin and self-densification, although small pits were observed (Figure 2I), likely due to the remnant of curcumin. The presence of curcumin in the WFC was confirmed by absorbance peaks in the FTIR spectra at 1,630 , 1,273 , and 965 cm^−1^, which are attributed to the C=O, C-O stretching, and C-H bending of alkene (Figure 1C), respectively. The XRD spectra of the WFC showed small peaks at 15.0° and 17.3° (Figure 1D), likely corresponding to the curcumin in crystals. The XRD pattern and the CrI of 74.7% for the WFC were similar to those of the WF, suggesting that the inclusion of curcumin did not alter the crystallinity and the structure of the cellulose. Therefore, the impregnation of curcumin resulted in the formation of WFC that retained the cellulosic structure that is similar to the WF without curcumin.

### Light transmittance and haze

**Figure 4:**
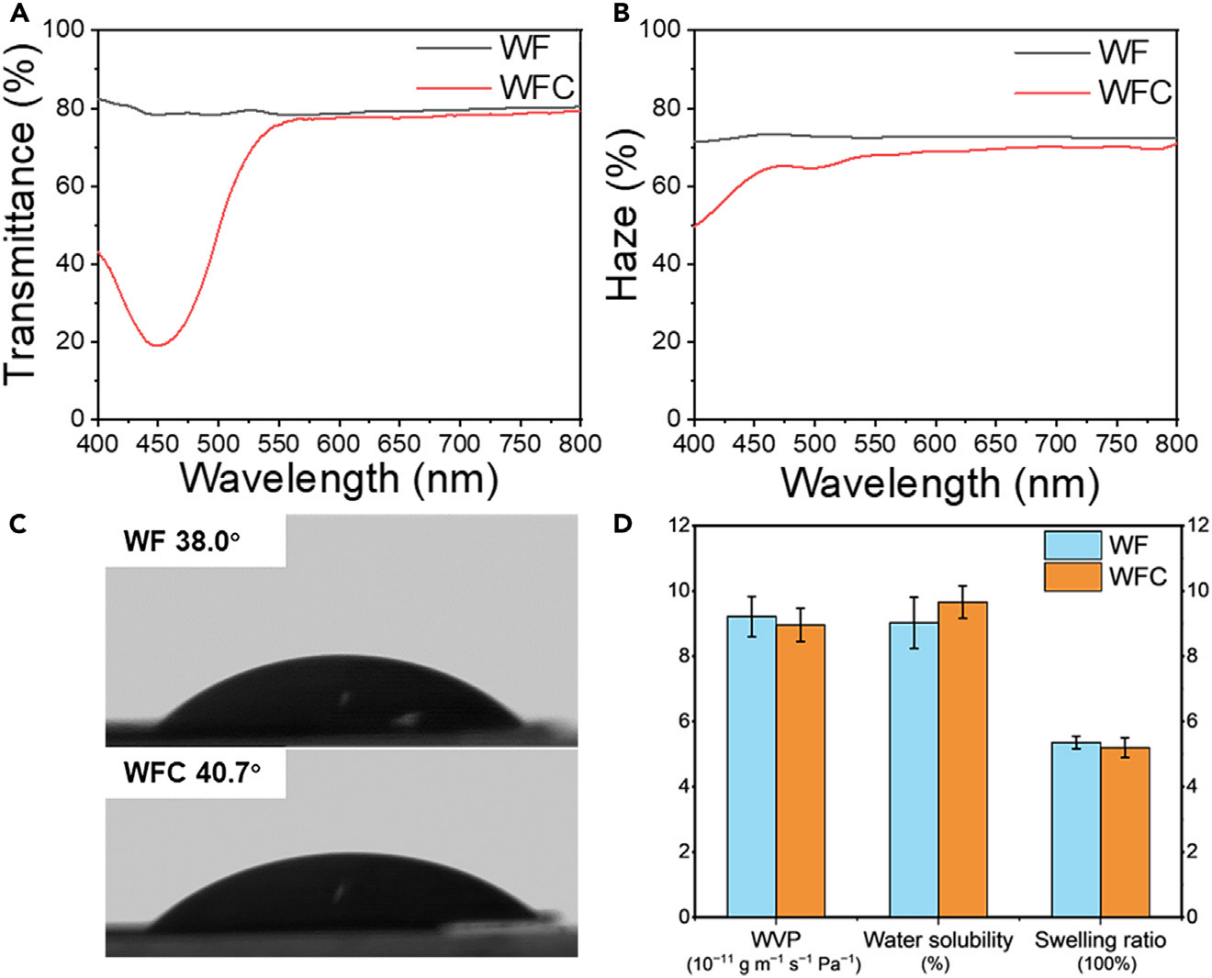
Optical properties and water sensitivity of wood film (WF) and wood film with curcumin (WFC) See also Figure S2. (A) Total light transmittance. (B) Haze. (C) Water contact angle. (D) Water vapor permeability (WVP), water solubility, and swelling ratio. Data are represented as mean ± SEM.

Optical properties are crucial for food packaging as they influence consumer purchasing decisions by allowing visual evaluation of product freshness and quality. Both the WF and WFC films produced in this study were visually transparent, and the text under the films was easily readable (Figure 2G). The WF film had an 80% total optical transmittance and a 72% total optical haze within the wavelength range of 400–800 nm (Figures 4A and 4B), which is comparable to that of the self-densified films reported previously.^25^ The removal of light-absorbing lignin from the WF was the primary contributor to the reduction in color. The WF and WFC were 50 μm in thickness, which is significantly thinner than the original wood (thickness = 1,500 μm). The air-drying densification process greatly minimized light scattering by narrowing the gap between cell walls, while the densified cellulose cell walls retained a uniform refractive index.^42^ The WFC exhibited a total light transmittance of 78% (Figure 4A), which showed a marginal decrease compared with the WF. A bending in the spectrum curve around 450 nm was observed in WFC, tentatively attributed to the strong absorption of curcumin.^43^ The haze of the WFC was 68% (Figure 4B), which was slightly lower than that of the WF. This reduction in haze was likely due to the penetration of curcumin into the material, leading to decreased light scattering within the material. The decreased haze of the WFC suggested that the incorporation of curcumin into the WF resulted in a reduced cloudiness and more uniform material, possibly because curcumin filled small voids and gaps within the wood structure.

As mentioned earlier, most transparent woods have been fabricated by filling delignified wood with external polymers having a refractive index comparable to cellulose. For example, transparent WFs were created by infiltrating the porous wood with prepolymerized methyl methacrylate, leading to high transmittance of 85% and a haze of 71%.^30^ In contrast, transparent WF developed in this study was fabricated through a simple densification process of delignified and oxidized wood, eliminating the complexity of polymer infiltration. Impregnation of curcumin followed by ambient densification was found to be efficient to produce a functionalized wood film with comparable level of transparency and haze. These findings suggest that the WF and WFC have promising potential for visually transparent food packaging windows.

### Water sensitivity

The WF produced in this study exhibited a water contact angle of 38.0° (Figure 4C), indicating its hydrophilic surface. The infiltration of curcumin slightly improved the film’s waterproof ability, revealed by an increased contact angle of 40.7°, possibly due to the hydrophobic aromatic constituents of curcumin. Water vapor permeability (WVP) is a crucial factor in food packaging materials, as it describes the film’s capability to transmit water vapor molecules. The WVP of the WF and WFC was 9.22 and 8.57 × 10^−11^ g m^−1^ s^−1^ Pa^−1^, respectively (Figure 4D). Both the WF and WFC contained abundant hydrophilic sites on their surfaces, such as hydroxyl and carboxyl groups, making them permeable to water vapor. Previous researchers observed that the WVP of cellophane films was between 2.22 and 7.12 × 10^−11^ g m^−1^ s^−1^ Pa^-1.^^44^ An increase in moisture content within the film may cause swelling, leading to the loss of closely packed structure and increased pore diffusion of water vapor.^45^ The solubility of the WF and WFC in water was found to be low at about 10%, with no significant difference (Figure 4D). Although cellulose is generally known to be insoluble in water due to the strong hydrogen bonding that occurs among cellulose molecules, the small solubility value of the film was due to the dissolving of remaining hemicellulose and fragmented cellulose in the film. The WF produced in this study had lower water solubility than the TEMPO CNF film reported to have a solubility of 18.4%.^46^ This difference was likely due to the preserved cell wall structure of the WF, including the well-aligned cellulose orientation, which promoted the proximity of cellulose fibers and strengthened hydrogen bonds within the WF film. The swelling ratios for WF and WFC were measured to be 5.36 and 5.20 (Figure 4D), respectively, which are higher than those of films made from nanocellulose, at around 2.^47^ This high swelling of the WF and WFC is probably attributed to the presumably lower crystallinity of wood fiber than nanocellulose (non-impregnable to water), so both adsorption and absorption of water on the WF and WFC likely occurred. Importantly, the overall structural integrity of the wood hydrogel was maintained throughout the swelling process. The WF demonstrated the capability to revert to a hydrogel state with a thickness of ∼1.5 mm when submerged in water (Figure S2). The alignment of the cellulose fibers in the hydrogel appeared to remain intact upon visual observation. Subsequent air-drying allowed the hydrogel to re-densify into a transparent film, suggesting the potential for recyclability of this WF.

### Mechanical properties

**Figure 5:**
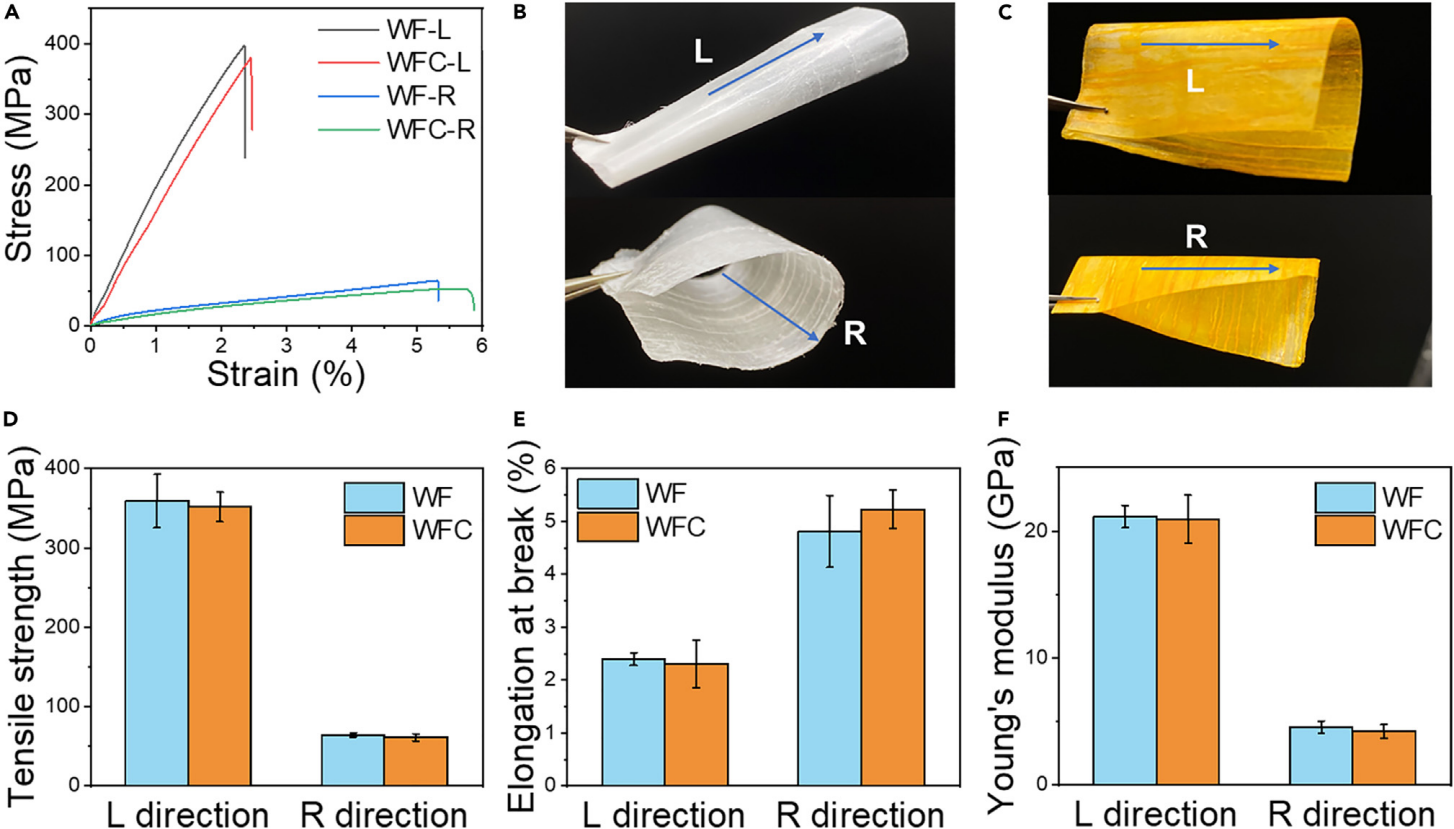
Evaluation of mechanical properties of wood film (WF) and wood film with curcumin (WFC) (A) Stress-strain curves of WF and WFC along the longitudinal (L) and radial (R) directions. (B) WF rolled in L and R directions. (C) WFC rolled in L and R directions. (D) Tensile strength. Data are represented as mean ± SEM. (E) Elongation at break. Data are represented as mean ± SEM. (F) Young’s modulus. Data are represented as mean ± SEM.

The WF and WFC exhibited remarkably strong mechanical strength (Figure 5A). The WF had a tensile strength of 359.03 MPa and Young’s modulus of 21.15 GPa along the longitudinal (fiber) direction, while along the radial direction, the tensile strength and Young’s modulus were 64.58 MPa and 4.56 GPa, respectively. Notably, the strength of the WF was substantially higher than that of natural wood, which typically had a tensile strength of 26.4 MPa and Young’s modulus of 1.6 GPa.^13^ These experimental findings were comparable with previous research in which a balsa WF prepared through self-densification demonstrated a tensile strength of 449.1 MPa and a Young’s modulus of 51.1 GPa in the fiber direction.^25^ The introduction of curcumin into the WF did not adversely impact its mechanical performance. The WFC exhibited a tensile strength of 351.75 MPa and a Young’s modulus of 20.94 GPa in the fiber direction (Figures 5D–5F). Both the WF and WFC showed super strong and anisotropic mechanical properties. Furthermore, the transparent WF produced in this study through a simple self-densification and polymer-free method exhibited almost four times higher strength than the polymer-infiltration method. For instance, transparent WF fabricated by infiltrating delignified balsa wood with poly(methyl methacrylate) showed a tensile strength of 90.1 MPa and Young’s modulus of 3.59 GPa.^30^ During the drying process without external polymers, the multilayer cell walls collapsed and were densely connected via hydrogen bonding. This resulted in the densified WF having more hydrogen bonding to interlock the cellulose fibers compared with the polymer-infiltrated wood or the original mesoporous natural wood, thus exhibiting stronger mechanical strength. The orientation of the natural cellulose fiber in wood endowed the film with anisotropic mechanical properties. The delignification and TEMPO-oxidization treatments employed in this work preserved the natural alignment of cellulose within the wood cell wall (Figure 2J). In the fiber direction, the orientated cellulose molecular chains are mainly covalently linked, showing high stretching resistance compared with the radial direction, where parallel cellulose bundles are mainly connected by intermolecular hydrogen bonding force.^48^ The film’s anisotropy was further evidenced by the elongation at break, which revealed inferior elongation in the fiber direction (2.40%) compared with the radial direction (4.81%). This difference arises from the significantly higher stretching resistance of the longitudinally aligned cellulose in comparison to the radial direction. Balsa wood exhibits a notable level of orientation, with cellulose microfibrils exhibiting a strong alignment along the fiber axis. The average microfibril angle of approximately 1.4°, determined via wide-angle X-ray scattering, provides evidence of this alignment.^49^ Manually controlling the orientation of extracted nanocellulose in a bottom-up method with external forces is usually not comparable with such ordered natural alignment, therefore, leading to noncomparable mechanical performance.^50^ The WF and WFC also showed high flexibility and toughness along both longitudinal and radial directions, as demonstrated by bending the films 180° without breaking (Figures 5B and 5C). The remarkable strength, acceptable flexibility, and ductility make the WF and WFC highly desirable for applications in food packaging.

### Application of WFC in sensing shrimp’s deterioration

**Figure 6:**
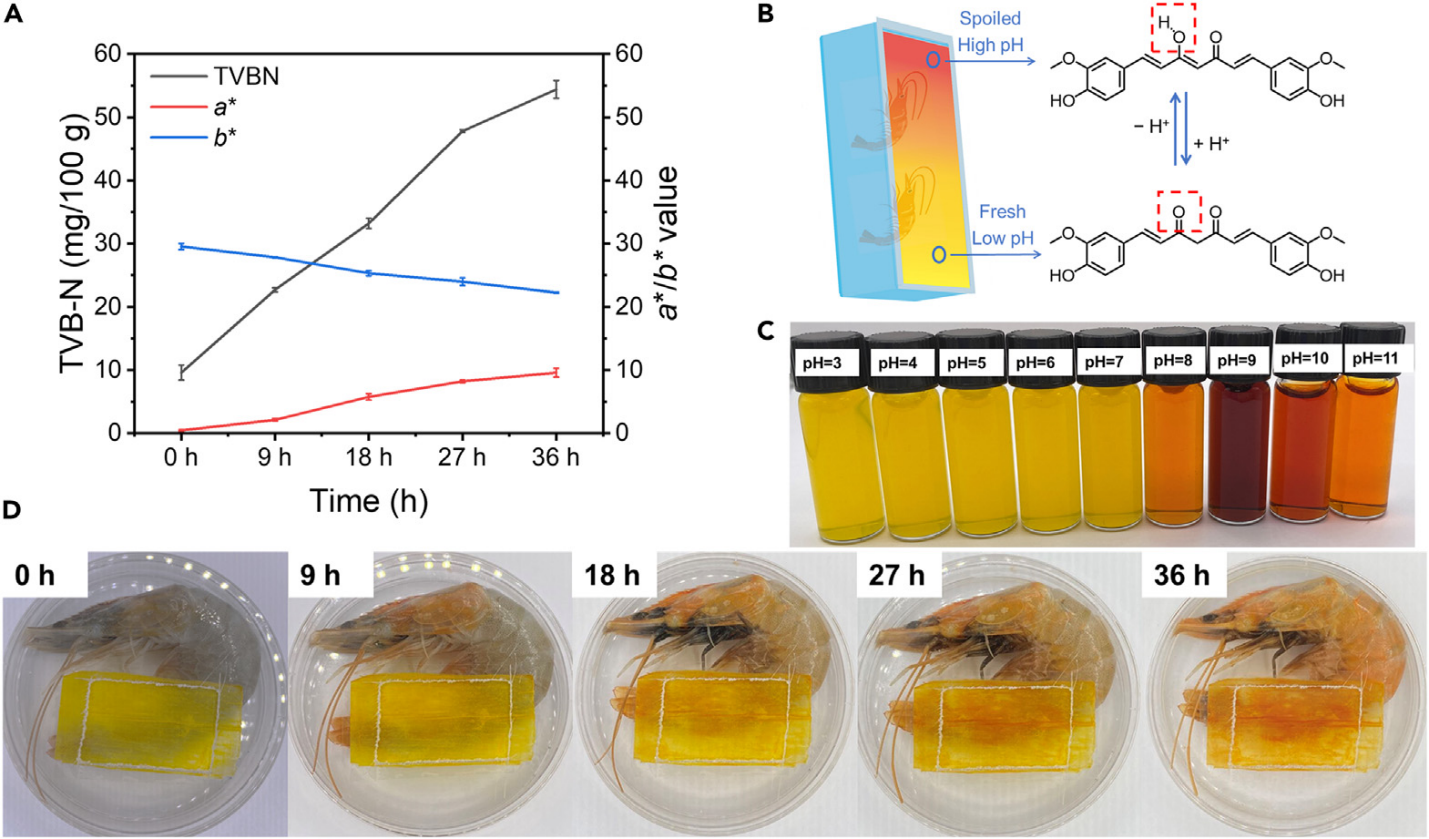
Application of wood film with curcumin (WFC) in sensing shrimp’s deterioration See also Figures S3–S6, and Table S1. (A) Total volatile basic nitrogen (TVB-N) content of shrimp and color parameters (a∗ and b∗) of WFC during shrimp spoilage. Data are represented as mean ± SEM. (B) Schematic illustration depicting the pH-responsive nature of WFC employed for monitoring shrimp freshness. (C) Color response of curcumin solution at pH 3–11. (D) Visual appearance of the shrimp packaged with WFC during storage.

Curcumin is a commonly used active ingredient in food as a spice and pigment, such as in curry powder. It has garnered increasing interest due to its potential health benefits as well as its antimicrobial and antioxidant activity.^43^^,^^51^ In addition, the color of curcumin is sensitive to pH, making it a useful pH-responsive indicator for monitoring food freshness.^43^ Curcumin is stable and exhibits a bright yellow color in acidic conditions (pH 1–7). Under alkaline conditions (pH > 7), the hydroxyl ions induce the tautomerization of the keto form to the enol form, causing the color to change to reddish (Figure 6B). The color of the curcumin solution ranged from bright yellow to dark red as the pH increases from 3 to 11 (Figure 6C). The ultraviolet-visible (UV-vis) spectra of curcumin solution showed an absorption peak at around 450 nm when the pH of the solution is between 3 and 7 due to the electron excitation from the π - π∗ transition of curcumin.^43^ With an increase in pH, a bathochromic shift occurred, resulting in the maximum absorbance wavelength being at 465 nm with higher intensity (Figure S3).

We evaluated the color response of the films to acidic and alkaline vapors. The WF was transparent, with *a∗* and *b∗* values close to 0 (Table S1 and Figure S4). The WFC showed initial bright yellow (*b∗* = 29.70). Upon exposure to HCl vapor, the film color remained yellow (*b∗* = 29.26) without significant change. However, exposure to NH_3_ vapor caused the film color to shift to a brownish-red hue (*a∗* = 14.34, *b∗* = 9.36), which was visually identifiable. This color-responsive function of curcumin and WFC allows for the potential design of a sensing window for food packaging. We further evaluated the release kinetics of curcumin from the WFC. The curcumin was released rapidly for the first 6 h, after which the concentration stabilized at approximately 12 μg/mL (Figure S5). Assuming that most of the curcumin has been released from the film, this is translated into approximately 0.8 mg curcumin loaded per gram dry weight of WFC. We are not concerned about the release behavior of curcumin in WFC because of the small amount of the window compared with the entire packaging box and the Generally Recognized as Safe (GRAS) grade of curcumin. Additionally, the released curcumin may be beneficial in preserving food quality owing to the inherent antioxidant properties of curcumin.^52^

The WFC was then used as a smart window to sense shrimp freshness. The film color shifted from bright yellow to brownish red as the storage time increased (Figure 6). The reddish color deepened after 18 h, as confirmed by the increasing *b*∗ value (Figure 6A). Simultaneously, the total volatile basic nitrogen (TVB-N) significantly increased during shrimp storage (Figure 6A). TVB-N is an indicator of meat spoilage that describes the alkaline nitrogenous compounds generated from protein degradation induced by microorganisms. During food storage, the growth and metabolic activities of microorganisms break down the protein component in food, generating organic acids such as acetic acid or volatile basic compounds such as trimethylamine. The limit of acceptability of TVB-N in shrimp is suggested to be 30 mg per 100 g.^53^ The volatile ammonium compounds interacted with water vapor in the packaging headspace, altering the pH within the WFC. This accumulation of ammonia gas accelerated the reddening of the film color due to the deprotonation of curcumin (Figure 6B). The WFC showed a sensitive color response to the shrimp spoilage, which could be visually detected as a smart window (Figure 6D). For comparison, we also tested the shrimp packaged with a window made of WF. It was observed that the color of the WF remained stable and unchanged throughout the shrimp spoilage process (Figure S6). Therefore, the WFC in this work exhibited potential as a practical tool for sensing the freshness and shelf life of food. We suppose that the WFC can be generally accepted as safe for food packaging applications according to the reported safety data of the major components—carboxylated cellulose and curcumin. TEMPO-oxidized cellulose has been documented to be non-toxic, as supported by *in vitro* cyto-genotoxicity studies.^54^ Additionally, curcumin has received the GRAS status from the Food and Drug Administration.^55^

### Conclusion

In conclusion, this study developed a polymer-free, strong, transparent, and pH-sensitive WF using a simple top-down approach. The functionalized WF, for the first time, was applied as a smart food packaging window by simply drying it in the air. The original balsa wood was delignified to remove the light-absorbing lignin, followed by TEMPO oxidation treatment to soften the wood structure and impart cellulose fibrils with carboxyl groups. The original hierarchical structure of the wood and the alignment of cellulose fibrils were retained in this process. IGC analysis revealed that delignification and TEMPO treatments reduced surface heterogeneity in the wood. Moreover, the ratio of $W_{Adh}^{Tot}$/ $W_{Coh}^{Tot}$ increased after treatments, indicating an enhanced water affinity that contributed to the cell wall collapse via capillary force. The WF exhibited an extraordinarily strong and anisotropic mechanical strength, and high optical light transparency because of the minimized light scattering at the wood cell pores after densification. The incorporation of curcumin allowed the WF to exhibit a pH-responsive color change, enabling the spoilage of shrimp to be monitored and visualized. This work demonstrates a simple and promising technique for applying wood-based film as a sustainable and smart food packaging material.

### Limitations of the study

This study was limited to the technology of fabricating transparent WF using relatively small size of wood samples. To facilitate the potential for large-scale production, future research dedicated to optimizing the reaction conditions for larger wood samples would be compelling. Moreover, a comprehensive evaluation of the film’s suitability, including but not limited to the film’s mechanical performance under varying humidity levels, and permeability to O_2_ and CO_2_ gases, using food packaging standards, is required.

## STAR★Methods

### Key resources table

**Table:** 

REAGENT or RESOURCE	SOURCE	IDENTIFIER
**Chemicals, peptides, and recombinant proteins**		

Sodium chlorite	Acros Organics	CAS#: 7758-19-2
Acetic acid	Fisher Scientific	CAS#: 64-19-7
Sodium hypochlorite	Fisher Scientific	CAS#: 7681-52-9
2,2,6,6- tetramethyl-1-piperidinyloxy	Sigma-Aldrich	CAS#: 2564-83-2
Sulfuric Acid	Fisher Scientific	CAS#: 7664-93-9
Curcumin	Sigma-Aldrich	CAS#: 458-37-7

**Other**		

Balsa wood (Ochroma pyramidale)	Guillow Inc	N/A
SEM	Zeiss Auriga	N/A
Microscope	Olympus BX51	N/A
XRD	PANalytical Empyrean	N/A
ATR-FTIR	PerkinElmer	N/A
IGC-SEA	Surface Measurement Systems Ltd.	N/A
Universal testing machine	Instron-5567	N/A
Drop shape analyzer	Kruss Easydrop	N/A
Haze measurement system	StellarNet	N/A
Colorimeter	CTI Portable Color Analyzer	N/A

### Resource availability

#### Lead contact

Further information and requests for resources and reagents should be directed to and will be fulfilled by the lead contact, Mi Li (mli47@utk.edu).

#### Materials availability

This study did not generate new unique reagents.

#### Data and code availability

(1) Data reported in this paper will be shared by the lead contact upon reasonable request.
(2) This study does not report any original code.
(3) Any additional information required to reanalyze the data reported in this paper is available from the lead contact upon reasonable request.

### Method details

#### Fabrication of transparent wood films

Balsa wood blocks (60 mm × 25 mm × 1.5 mm) were first delignified by soaking them in a 2% NaClO_2_/acetate buffer (pH 4.6) at 80°C for 12 h, followed by rinsing thoroughly with distilled water. The delignified wood was then submerged and oxidized in a 200 mL solution mixture (pH 6.8) containing 0.4 mM TEMPO, 50 mM NaClO_2_, and 4 mM NaClO. The oxidation reaction proceeded at 60°C for 48 h without agitation. Subsequently, one delignified and TEMPO-oxidized wood sample was gently rinsed with distilled water three times and air-dried under ambient conditions (25°C, 30% RH) for 48 h to obtain transparent wood films (WF). Another piece of delignified and TEMPO-oxidized wood was rinsed and freeze-dried to produce wood aerogel. The TEMPO-oxidized wood was immersed in a curcumin solution (1 mg/mL, with an ethanol to water ratio of 1:4 *v/v*), vacuum impregnated for 30 min three times, and then air-dried in ambient conditions for 48 h to obtain functionalized wood-based transparent film WFC.

#### Characterizations of WF and WFC

The concentration of acid-insoluble lignin was measured following the NREL standard method.^56^ The biomass sample (300 mg, dry basis) was first hydrolyzed with 72 wt % sulfuric acids at 30°C for 1 h. The hydrolysis was continued in an autoclave at 121°C for 1 h using 4 wt % sulfuric acids, obtained by diluting the initial solution with the appropriate amount of distilled water. The hydrolysate was then filtered and dried. Lignin content was determined by dividing the dry weight of lignin by the dry weight of biomass sample.

The concentration of carboxylate groups in the wood sample was determined using conductometric titration, following a previously established method.^57^

The micromorphology of delignified wood, TEMPO-oxidized wood aerogel, WF, and WFC was characterized using scanning electron microscopy. Cross-sectional and longitudinal views of WF and WFC were obtained by fracturing the films after submerging them in liquid nitrogen.

A microscope, equipped with polarizer and analyzer filters and a 10× objective lens, was employed to characterize wood film. During imaging, the polarizer and analyzer filters were rotated by 90° to capture the image of the sample.

The crystalline structure of WF and WFC was determined using an X-ray diffraction (XRD) diffractometer at 40 kV and 40 mA. Samples were scanned with a step size of 0.02° and a scanning rate of 4° min^−1^. The crystallinity index (CrI) was calculated using the following equation^58^:

$$CrI\;\left(\%\right)=\left(I_{002}-I_{am}\right)/I_{002}\times 100$$

where I_002_ is the maximum XRD intensity at 2θ = 22.6°, and I_am_ is the intensity of the amorphous region at 2θ = 18.9°.

The functional groups of WF and WFC were analyzed using attenuated total reflectance-Fourier transform infrared (ATR-FTIR) spectroscopy. The spectra were obtained with 16 scans in a spectral range of 400–4000 cm^−1^ at a resolution of 4 cm^−1^.

Surface energy analyses and Brunauer–Emmett–Teller (BET) surface area were measured using an Inverse Gas Chromatography-Surface Energy Analyzer (iGC-SEA).^59^ Each sample (20–50 mg) was packed into a 4 mm chromatographic column and conditioned under a nitrogen gas flow for 2 h. The dispersive components of the surface energy were measured using n-alkanes probes, which included octane, nonane, decane, and undecane. The specific (acid-base) components of the surface energy were ascertained using ethyl acetate and dichloromethane. All measurements were carried out under controlled conditions (30°C, 0% RH). The Brunauer-Emmett-Teller (BET) specific surface areas were determined by calculating the isotherm of octane with a pressure ratio (P/P_0_) ranging between 0.05 and 0.35. The data were analyzed using the advanced SEA Analysis Software.

The tensile properties of the transparent wood films were measured with a universal testing machine. Each sample was cut into a strip (50 mm × 5 mm) for testing. The sample strip was stretched at a tension speed of 5 mm/min.

The water contact angle of films was determined with a drop shape analyzer, recorded at 30 s. The water solubility of films was assessed by immersing 0.5 g of film in water and shaking continuously at 150 rpm at room temperature for 24 h, followed by drying at 105°C to achieve a constant weight. Solubility was calculated as the weight loss ratio after dissolution compared with the initial weight. The water vapor permeability (WVP) of the films was determined using a gravimetric method. The wood film was sealed on top of a 50 mL plastic tube containing 40 g of dry silica (0% RH). The tubes were placed in a desiccator containing distilled water (100% RH) at 25°C. The weight gain of the tube was measured every 24 h for 3 days. Swelling ratio was measured by immersing accurately weighed (∼0.2 g) WF or WFC in 30 mL of distilled water. The samples were left to soak for 30 min at room temperature without agitation. After taking the sample out of the water, the excess water was gently removed from the samples using filter paper. The swelling ratio was then calculated by taking the ratio of the film’s wet weight to its original dry weight.

Total transmittance and haze were measured using a spectrometer equipped with an integrating sphere, following the ASTM 1003-13 standard.^60^ The white reference (*T*_*1*_) was recorded by directing the light beam into the integrating sphere. The sample was then placed in front of the integrating sphere to obtain the signal spectrum (*T*_*2*_). The total light transmittance was calculated as *T*_*1*_/*T*_*2*_. The integrating sphere was capped with a light trap to acquire the diffuse reference (*T*_*3*_), and the diffuse transmittance (*T*_*4*_) was collected by positioning the sample in front of the integrating sphere with the light trap in place. Haze was calculated as:

$$\text{Haze}=\left(\frac{T_{4}}{T_{2}}-\frac{T_{3}}{T_{1}}\right)\times 100\%$$

The sensitivity of films to volatile gases was assessed by exposing the film (60 mm × 25 mm) to the headspace of a sealed tube containing 15 mL of either 1 M hydrochloric acid or 1 M ammonium hydroxide at 25°C for 10 min.

#### Release of curcumin in WFC

The WFC film was immersed in an ethanol solution to achieve a final concentration of 1.5% (*w/v*) and was agitated at 150 rpm at room temperature. The curcumin concentration was measured at predetermined time intervals using a UV-Vis spectrometer at 425 nm.

#### Application of the WFC for sensing shrimp’s deterioration

Fresh shrimp was obtained from a local market. A rectangular window hole (45 mm × 20 mm) was cut out from the lid of a 90 mm Petri dish. The WFC film was mounted to the hole from the inside to seal the window. The WFC-mounted Petri dish lid was then used to cover the dish containing the shrimp sample, allowing for monitoring of shrimp deterioration. The Petri dish lid was further sealed with parafilm and stored at 25°C. Samples were taken every 9 h to measure shrimp total volatile basic nitrogen (TVB-N) and film color. TVB-N was measured using the Kjeldahl method.^61^ The color parameters (*L∗*, *a∗*, and *b∗*) of the WFC window were measured with a colorimeter, and the appearance of the packaging system was recorded using a digital camera. The *a∗* value represents the position between red and green (−*a∗* for greenness and +*a∗* for redness), the *b∗* value represents the position between yellow and blue (−*b∗* for blueness and +*b∗* for yellowness), and the *L∗* value represents color lightness. Total color difference (*ΔE*) was calculated using the following equation:

$$\Delta E=\sqrt{{\left(L^{*}-L_{0}\right)}^{2}+{\left(a^{*}-a_{0}\right)}^{2}+{\left(b^{*}-b_{0}\right)}^{2}}$$

where *L*_*0*_, *a*_*0*_, and *b*_*0*_ are the color values of the white plate (*L*_*0*_ = 98.93, *a*_*0*_ = 0.01, and *b*_*0*_ = 0.33).
