# Supplementary material for: Ambient-densified and polymer-free transparent wood film for smart food packaging window
Source: iScience. 2023 Nov 15;26(12):108455. doi: 10.1016/j.isci.2023.108455 (PMC10709124; doi:10.1016/j.isci.2023.108455)
Supplement: Document S1. Figures S1–S6 and Table S1 [file mmc1.pdf]

iScience, Volume *26*

Supplemental information

Ambient-densified and polymer-free transparent wood film for smart food packaging window

Kailong Zhang, Isaac Sutton, Micholas Dean Smith, David P. Harper, Siqun Wang, Tao Wu, and Mi Li

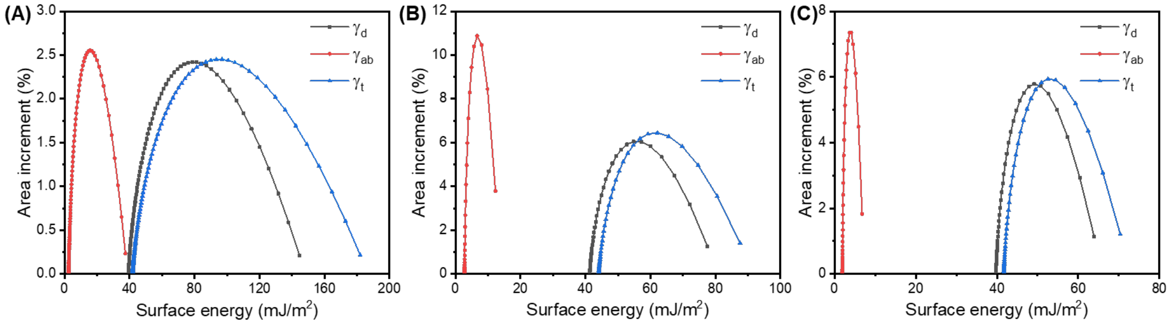

Figure S1. Distribution of the dispersive (γ_S^D), specific (γ_S^{AB}), and total (γ_S^{Tot}) surface energy. (A) original wood. (B) Delignified wood. (C) TEMPO-oxidized wood. Related to Figure 3.

The total energy (γ_S^{Tot}) of solid surfaces is composed of dispersive (γ_S^D) and specific (acid-base, γ_S^{AB}) components. The dispersive surface energy (γ_S^D) can be calculated by using the retention of liquid n-alkane probes at infinite dilution. This is because alkanes have no acid-base interaction. [1] The specific component (γ_S^{AB}), originates from the interactions based on solids' electron acceptor (Lewis acid) and electron donor (Lewis base) properties. Dichloromethane and ethyl acetate, due to their inherent electron donor and acceptor attributes, can serve as two monopolar acidic and basic probes, respectively. [2]

Total work of cohesion (W_{Coh}^{Tot}) and adhesion (W_{Adh}^{Tot}) can be calculated with the following equation. [3]

$$W_{Coh}^{Tot} = 2(\gamma_S^D + \sqrt{\gamma_S^{AB+} \cdot \gamma_S^{AB-}} + \sqrt{\gamma_S^{AB-} \cdot \gamma_S^{AB+}}) \quad \textbf{Equation S1}$$

$$W_{Adh}^{Tot} = 2(\sqrt{\gamma_S^D \cdot \gamma_W^D} + \sqrt{\gamma_S^{AB+} \cdot \gamma_W^{AB-}} + \sqrt{\gamma_S^{AB-} \cdot \gamma_W^{AB+}}) \quad \textbf{Equation S2}$$

where γ_S^{AB+} and γ_S^{AB-} are the acid and base components of the specific surface energy γ_S^{AB} of the solid. γ_W^D is the dispersive surface energy of water, while γ_W^{AB+} and γ_W^{AB-} are the acid and base components of water's specific surface energy. The values for γ_W^D, γ_W^{AB+} and γ_W^{AB-} are 21.8, 25.5, and 25.5 mJ/m^2, respectively.

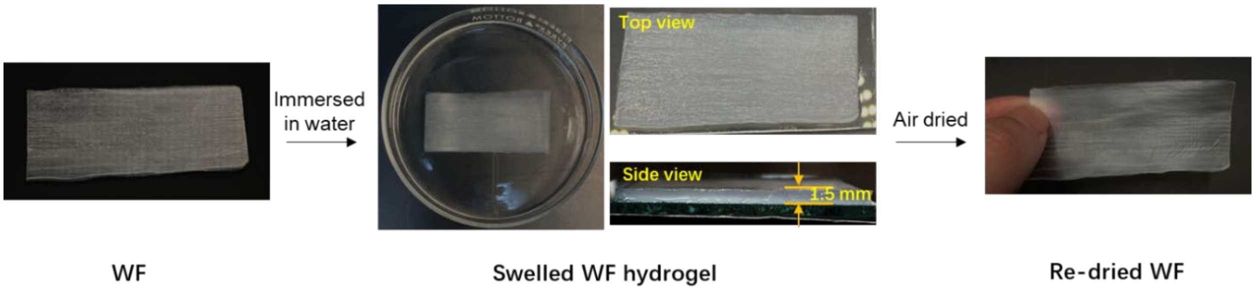

Figure S2. Water-induced swelling of WF after 30-minute immersion and subsequent re-densification upon air drying. Related to Figure 4.

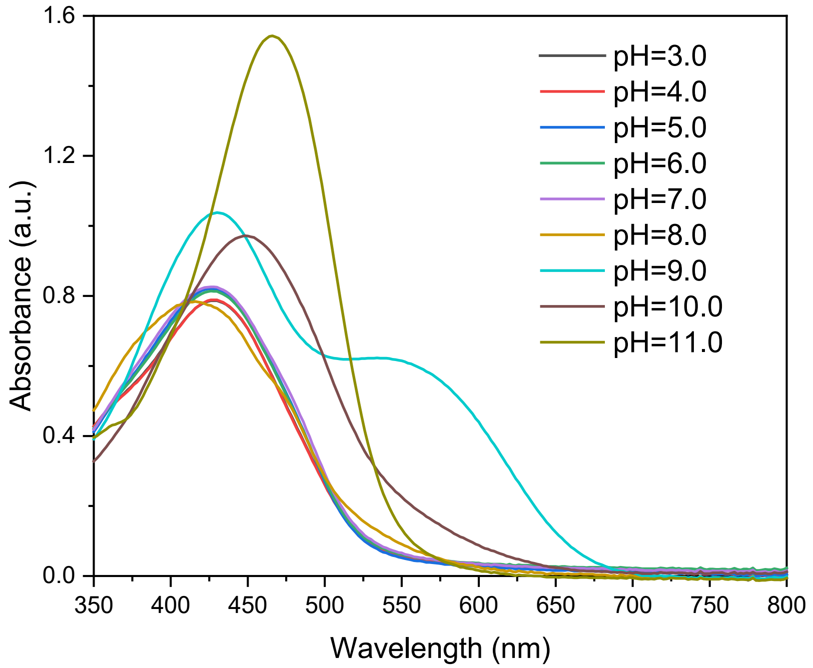

Figure S3. UV–Vis spectra of curcumin solution at pH 3-11. Related to Figure 6.

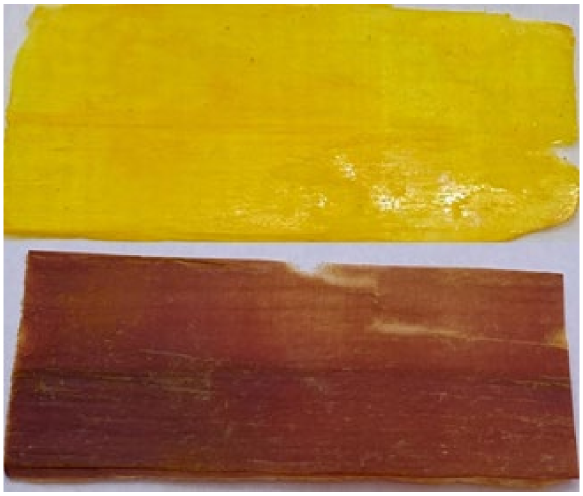

Figure S4. Visual appearance of WFC after exposure to HCl (top) and NH_3 (bottom). Related to Figure 6.

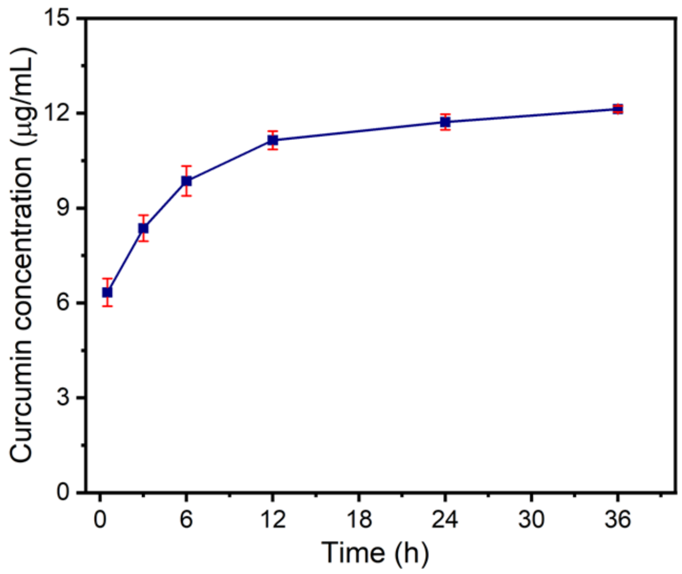

Figure S5. Release behavior of curcumin loaded in WFC. Data are represented as mean ± SEM. Related to Figure 6.

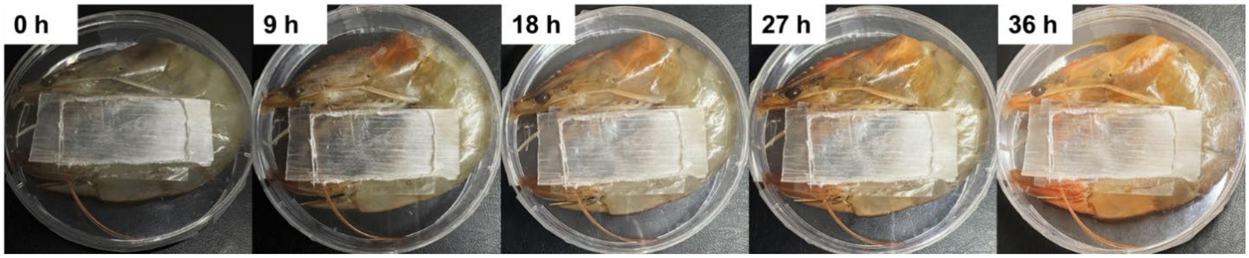

Figure S6. Visual appearance of the shrimp packaged with WF during storage. Related to Figure 6.

Table S1. Color parameters of WF, WFC, and WFC exposed in HCl and NH_3. Data are represented as mean ± SD. Related to Figure 6.

	*L**	*a**	*b**	*ΔE*[*]
WF	59.39±0.92	0.15±0.06	-0.92±0.34	39.59±0.91
WFC	60.56±1.15	5.43±0.71	29.70±0.94	48.66±0.80
WFC-HCl	56.93±1.28	0.53±0.24	29.66±0.80	51.26±0.91
WFC-NH_3	46.12±0.71	14.34±1.13	9.36±0.24	55.50±0.42

[*] *ΔE* is the Euclidean distance between two color coordinates, representing the distance between two points in a color space.

References

1. Schultz, J., and Lavielle, L. (1989). Interfacial properties of carbon fiber—epoxy matrix composites. In Inverse Gas Chromatography (ACS Publications), pp. 185–202.
2. Mohammadi-Jam, S., and Waters, K. (2014). Inverse gas chromatography applications: A review. Adv. Colloid Interface Sci. *212*, 21-44.
3. van Oss, C.J., Chaudhury, M., and Good, R.J. (1987). Monopolar surfaces. Adv. Colloid Interface Sci. *28*, 35-64.
